# The Efficacy of Graded Motor Imagery and Its Components on Phantom Limb Pain and Disability: A Systematic Review and Meta-Analysis

**DOI:** 10.1080/24740527.2023.2188899

**Published:** 2023-05-17

**Authors:** Katleho Limakatso, Aidan G. Cashin, Sam Williams, Jack Devonshire, Romy Parker, James H. McAuley

**Affiliations:** a School of Health Sciences, Faculty of Medicine and Health, University of New South Wales, Sydney, Australia; b Centre for Pain IMPACT, Neuroscience Research Australia (NeuRA), Sydney, Australia; c Pain Management Unit, Department of Anaesthesia and Perioperative Medicine, University of Cape Town, Cape Town, South Africa

**Keywords:** Phantom limb pain, mirror therapy, imagined movements, left/right judgments, graded motor imagery, amputation

## Abstract

**Introduction:**

Graded Motor Imagery (GMI) is a non-invasive and inexpensive therapy used to treat Phantom Limb Pain (PLP) by sequentially activating motor networks in such a way that movement and pain are unpaired. The objective of this systematic review was to critically appraise relevant data on the efficacy of GMI and its components for reducing PLP and disability in amputees.

**Methods:**

We searched 11 electronic databases for controlled trials investigating GMI and its components in amputees with PLP from inception until February 2023. Two reviewers independently screened studies and extracted relevant data. Study-level data were entered using the inverse variance function of the Review Manager 5 and pooled with the random effects model.

**Results:**

Eleven studies with varying risk of bias were eligible. No eligible study considered left/right judgement tasks in isolation. Studies showed no effect for imagined movements, but positive effects were seen for GMI [weighted mean difference: -21.29 (95%CI: -31.55, -11.02), I^2^= 0%] and mirror therapy [weighted mean difference: -8.55 (95%CI: -14.74, -2.35, I^2^= 61%]. A comparison of mirror therapy versus sham showed no difference [weighted mean difference: -4.43 (95%CI: -16.03, 7.16), I^2^= 51%].

**Conclusion:**

Our findings suggest that GMI and mirror therapy may be effective for reducing PLP. However, this conclusion was drawn from a limited body of evidence, and the certainty of the evidence was very low. Therefore, rigorous, high-quality trials are needed to address the gap in the literature and inform practice.

## RÉSUMÉ

**Contexte:** L’imagerie motrice graduelle (IMG) est un traitement non invasif et peu coûteux utilisé pour traiter la douleur du membre fantôme par activation séquentielle des réseaux moteurs de manière à ce que le mouvement et la douleur soient dissociés. L’objectif de cette revue systématique était d’évaluer de manière critique les données sur l’efficacité de l’IMG et de ses composantes pour réduire la douleur du membre fantôme et l’invalidité chez les amputés.

**Méthodes:** Nous avons effectué des recherches dans 11 bases de données électroniques afin d’y repérer des essais contrôlés portant sur l’utilisation de l’IMG et de ses composantes auprès des amputés atteints de douleur du membre fantôme depuis le début jusqu’en février 2023. Deux évaluateurs indépendants ont examiné les études et extrait les données pertinentes Les données au niveau de l’étude ont été saisies à l’aide de la fonction de variation inverse de Review Manager 5 et regroupées selon un modèle à effets aléatoires.

**Résultats:** Onze études présentant un risque de biais variable ont été retenues. Aucune étude admissible ne se penchait sur les tâches de jugement gauche/droite de manière isolée. Les études n’ont montré aucun effet pour les mouvements imaginés, mais des effets positifs ont été observés pour l’IMG [différence moyenne pondérée : -21,29 (IC à 95 % : -31,55, -11,02), I^2^ = 0 %] et la thérapie miroir [différence moyenne pondérée: -8,55 (IC 95% : -14,74, -2,35, I^2^ = 61%]. La comparaison de la thérapie miroir à une thérapie factice n’a montré aucune différence [différence moyenne pondérée : -4,43 (IC à 95 % : -16,03, 7.16), I^2^ = 51 %].

**Conclusion:** Nos résultats indiquent que l’IMG et la thérapie miroir peuvent être efficaces pour réduire la douleur du membre fantôme. Cependant, cette conclusion a été tirée à partir d’un ensemble limité de données probantes, et la certitude de ces dernières était très faible. Par conséquent, des essais rigoureux et de haute qualité sont nécessaires pour combler les lacunes dans la littérature et éclairer la pratique.

## Introduction

Phantom limb pain (PLP) is a common postamputation syndrome characterized by painful sensations in the missing part of the amputated limb. A recent systematic review revealed a PLP incidence of 82% within the first year of undergoing an amputation and a lifetime prevalence of 87%.^1^ PLP is associated with psychological distress,^2^ disability,^3,4^ and poorer health-related quality of life (HRQoL).^5,6^ PLP remains poorly understood and difficult to treat.^7^

Treatments recommended for PLP are marginally effective at best and no more effective than placebo. There is systematic review evidence that pharmacological treatments are ineffective: memantine (30 mg/day for 4 days), gabapentin (2.4 g/day for 6 weeks), and amitriptyline (10–125 mg/day for 6 weeks) showed no benefit over placebo.^8,9^ Moreover, a review of the recent literature showed that of the six treatments investigated (targeted muscle reinnervation, repetitive transcranial magnetic stimulation, imaginal phantom limb exercises, mirror therapy, virtual reality and augmented reality therapies, eye movement desensitization and reprocessing therapy), none were more effective than the control.^10^ The lack of effectiveness of these interventions suggests that they do not effectively target the mechanisms that underlie PLP.

Neuroimaging evidence has linked PLP to cortical reorganization of the sensorimotor cortex, in which the cortical area that previously represented the missing limb comes to represent other body parts.^11,12^ However, Makin et al. challenged this association between PLP and cortical reorganization by consistently revealing preserved cortical representation and function of the missing limb in amputees with PLP.^13–15^ More recently, Ortiz-Catalan argued that PLP is purportedly driven by the stochastic entanglement of somatosensory–motor and pain networks resulting from somatosensory and motor deprivation.^16^ This therefore suggests that phantom motor execution exercises providing motor and somatosensory feedback, such as mirror therapy, may be effective in reducing PLP.^17^

Mirror therapy was proposed as a treatment for PLP because it purportedly addresses a theorized mismatch between motor command and sensory feedback.^18^ Mirror therapy involves positioning a mirror in the sagittal plane of the body and moving the intact limb while viewing its reflection in the mirror, such that the reflection appears to be the missing limb. Mirror therapy has also been used as the third component of a three-phase graded motor imagery (GMI) program, which was developed to progressively target cortical motor networks in people with complex regional pain syndrome (CRPS).^19^ GMI has systematic review evidence supporting its use in patients with CRPS.^20,21^ The similarities between the cortical changes seen in patients with CRPS and PLP suggest that the GMI program in its entirety could be a viable treatment for PLP.^22^

Single studies have investigated mirror therapy, the other components of GMI (left/right judgment exercises and imagined movements), and the full GMI program for alleviating PLP, but to our knowledge there has been no recent attempt to systematically synthesize this literature. We therefore aimed to gather and critically appraise all relevant literature regarding the efficacy of the three components of GMI and the entire GMI program for reducing PLP to guide ongoing research and clinical practice.

## Methods

This review was developed using the Cochrane methodology for systematic reviews^23^ and has been reported following the PRISMA 2020 statement.^24^ The protocol of this review has been registered on PROSPERO (Ref. No. CRD42016036471) and published elsewhere.^25^

### Identification of Studies

We used a customized search strategy (Supplementary file 1) to search the following electronic databases: PubMed, Cochrane Central Register of Controlled Trials, Medline (via Ebscohost), PsychINFO (via Ebscohost), Physiotherapy Evidence Database, Scopus, Cumulative Index to Nursing and Allied Health Literature (via Ebscohost), Literatura Latino Americana em Ciências da Saúde, Database of Abstracts of Reviews of effects in the Cochrane Library, Africa-Wide Information (via Ebscohost), and Web of Science. In addition, we searched clinicaltrials.gov, Pactr.gov, and the European Union clinical trials register for ongoing research. Electronic databases and clinical registries were searched from their inception until February 2023.

To identify gray literature, we searched OpenGrey and contacted experts to seek published, unpublished, and ongoing trials that may be eligible for inclusion.

### Eligibility Criteria

Studies were eligible for inclusion if they were randomized controlled trials, included adults (≥18 years) with chronic (≥3 months) PLP after amputation of an upper or lower limb, and compared GMI or one of its components to a control treatment. GMI was defined as treatment provided in order of left/right judgments, imagined movements, and mirror therapy. This ordered application of the components is thought to sequentially activate cortical premotor and motor networks and has been shown to produce a superior effect compared to the unordered GMI program.^19^ Studies had to be published in the English language and needed to report at least one outcome of interest. If studies included participants with other pathologies or measured other outcomes, only the data relevant to the question of this review were extracted and used.

### Screening and Study Selection

Two reviewers (S.W. and J.D.) independently screened titles and abstracts of studies retrieved from the literature search in duplicate. We retrieved full-length records of those studies deemed eligible and screened these again to confirm inclusion. Disagreements were resolved through discussion or, when necessary, consultation of a third independent reviewer (K.L.). When further information was required to confirm eligibility, we contacted authors up to four times within a 2-week period.^26^ We used Cohen’s kappa to determine the measure of agreement between reviewers as either minimal (0–0.39), weak (0.40–0.59), substantial (0.60–0.79), or strong (0.80–0.90).^27^

### Outcomes

The primary outcome of interest was a change in PLP severity assessed by a 0 to 100 mm visual analogue scale (VAS) or 11-point numerical rating scale. The secondary outcomes were disability, HRQoL, adverse effects, psychosocial function, and patient global impression of change.

### Data Extraction

Two reviewers (K.L. and J.D.) independently extracted data from included studies using a piloted customized sheet. Extracted data included the study characteristics (e.g., design, setting, exclusion/inclusion criteria, number of participants per group), participant characteristics (e.g., age, gender, amputation type, comorbidities), treatment characteristics (e.g., description, duration, frequency), follow-up period (weeks), number of participants lost to follow-up, adverse effects, and outcome measures (baseline, after intervention, and follow-up results on outcome measures). The two reviewers (K.L. and J.D.) compared the results and resolved disagreements concerning data extraction by discussion.

### Assessment of Risk of Bias in Included Studies

Two reviewers (K.L. and S.W.) independently assessed the risk of bias of each included study using a customized risk of bias assessment guide (Supplementary file 2) informed by the Cochrane risk of bias tool.^28^ The tool assessed the risk of bias across the domains of random sequence generation, allocation concealment, blinding of participants and outcome assessment, incomplete outcome data, selective outcome reporting, and other sources of bias. Studies received an overall summary risk of bias score of “high risk” if the study was scored as high risk for any individual category, “low risk” if it was scored as low risk for every category, and “unclear” if it was scored as unclear for any category and did not score as high risk for any category. All disagreements were resolved by discussion.

### Certainty of the Evidence

Two reviewers (K.L. and J.D.) independently assessed the certainty of the evidence for each analysis using the GRADE system.^29^ We downgraded the certainty of evidence if a serious flaw was present in the domains of risk of bias, inconsistency, imprecision, indirectness, and publication bias. The certainty of evidence was initially classified as high and then as moderate, low, or very low certainty. All disagreements were resolved by discussion.

### Data Analysis

Data were analyzed using Review Manager 5.^30^ We pooled the results in a meta-analysis using the random effects inverse variance model.^23^ We pooled studies comparing GMI with routine care, mirror therapy with control treatments, and mirror therapy with sham (covered mirror therapy). We calculated the weighted mean difference with a 95% confidence interval (CI) to determine between-group differences in outcomes for each analysis. A weighted mean difference of >10 mm (on a 0–100 mm VAS) with a 95% CI lower limit of ≥10 mm was considered clinically significant.^31^ We converted the scores of the two studies^6,32^ that assessed pain using a 0 to 10 scale to a 0 to 100 scale by multiplying the mean and standard deviation with the range of the new scale.^33^ Funnel plots were generated to assess for possible publication bias whenever possible. We assessed statistical heterogeneity using the *I*^2^ statistic and rated the level of heterogeneity as low (0%–25%), moderate (>25%–50%), or high (>50%).^23^ An improvement of ≥20% in HRQoL was considered clinically meaningful in accordance with anchor- and distribution-based methods for assessing the minimum clinically important change in HRQoL.^34^ In a three-arm study, we reused data from the experimental group such that we had two between-group comparisons. Statistical significance was set at *P* < 0.05 for all analyses.

## Results

**Figure 1.**
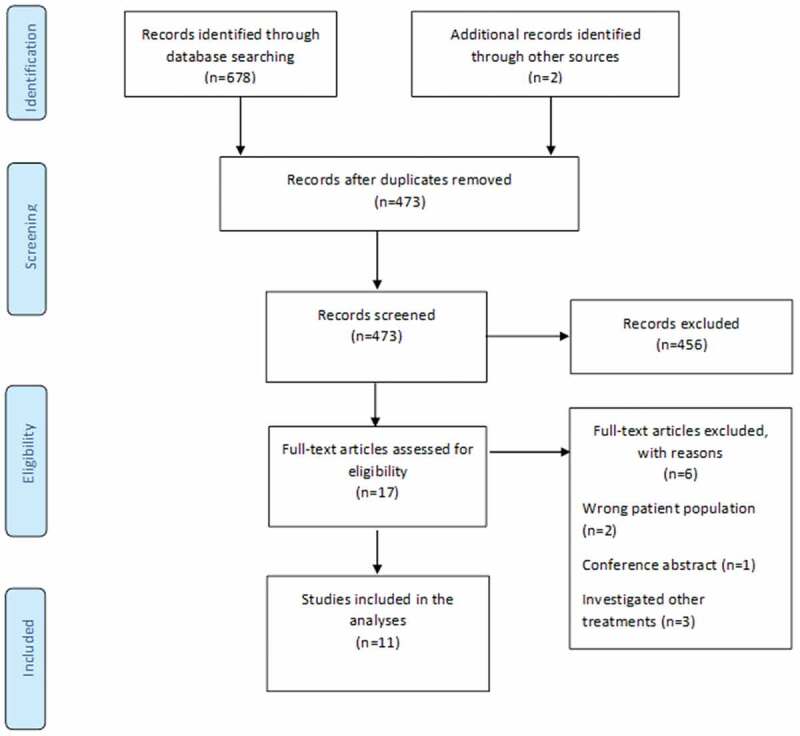
The PRISMA flowchart.

The initial literature search yielded 473 studies after removal of duplicates. Following title and abstract screening, 17 studies proceeded to full-text screening. Eleven studies were eligible and were included in this review (Fig. 1). Two studies^6,35^ investigated GMI (versus routine care) and four studies^36–39^ investigated mirror therapy (versus sham). Four other studies^32,40–42^ investigated mirror therapy (versus imagined movements, phantom movement exercises, tactile training, or transcutaneous electrical nerve stimulation), and two clinically heterogenous studies^37,43^ investigated imagined movements (versus direct limb observation and mirror therapy). No study examined left/right judgment exercises as a stand-alone treatment. One three-arm study^37^ compared mirror therapy with covered mirror therapy and imagined movements. Therefore, 12 between-group comparisons were included in our analysis. The screening of titles and abstracts and full-text articles reflected strong (kappa = 0.85) and substantial (kappa = 0.76) agreement between reviewers, respectively.

The 11 studies provided data from a total of 373 participants (297 male, 76 female), of whom 37 reported PLP in an upper limb and 336 reported PLP in a lower limb. Further details of participants’ characteristics are provided in Table 1. Treatment parameters used in the different studies varied: each treatment session lasted between 10 and 30 min, treatment frequency ranged between one and seven sessions per week, and the total duration of interventions ranged between 1^36^ and 42 days.^35^ Three studies^35,40^ reported follow-up measures at 6 months after treatment. Treatment details are summarized in Supplementary file 3.

**Table 1.** Characteristics of patients in included studies.

Study	Country of study	Total number of participants	Age, mean ± SD (experimental)	Age, mean ± SD (control)	Type of amputation: LL/UL (experimental)	Type of amputation: LL/UL (control)	Sex: M/F (experimental)	Sex: M/F (control)
Anaforoğlu et al.^40^	Turkey	40	32.60 ± 7.39	29.60 ± 6.87	20/0	20/0	12/8	13/7
Brodie et al.^36^	United Kingdom	80	54	57	41/0	39/0	35/6	28/11
Chan et al.^37^	United States	18	—	—	6/0	12/0	0/6	0/12
Finn et al.^38^	United States	15	—	—	0/9	0/6	9/0	6/0
Limakatso et al.^6^	South Africa	21	60 ± 12	62 ± 11	11/0	9/1	8/3	8/2
Moseley^35^	Australia	9	41 ± 14	41 ± 14	3/2	2/2	2/3	2/2
Ol et al.^41^	Cambodia	30	57.5 ± 6.0	52.0 ± 7.0	15/0	15/0	—	—
Ramadugu et al.^39^	India	64	—	—	—	—	32/0	32/0
Rothgangel et al.^32^	The Netherlands	50	59.7 ± 16.1	61.0 ± 15.2	26/0	24/0	21/5	17/7
Tilak et al.^42^	India	26	42.62 ± 10.69	36.38 ± 9.55	9/4	10/3	12/1	11/2
Tung et al.^43^	United States	20	—	—	9/0	11/0	9/0	11/0

LL = lower limb; UL = upper limb.

Nine of the 11 included studies used a 100 mm VAS to assess pain by self-report. One study assessed HRQoL using the VAS (0 = *worst imaginable health state* to 100 = *best imaginable health state*) of the EuroQol EQ-5D-5L.^6^ One study^35^ also assessed PLP-related disability as a secondary outcome using a patient-specific task-related numerical rating scale.^44^ In that study, the participants rated their ability to perform five self-selected activities on a Likert-type scale (0–10 VAS: 0 = *completely unable to perform*; 10 = *able to perform normally*). No studies reported data on psychosocial outcomes, adverse effects, and patient global impression of change.

### Risk of Bias Assessment

**Figure 2.**
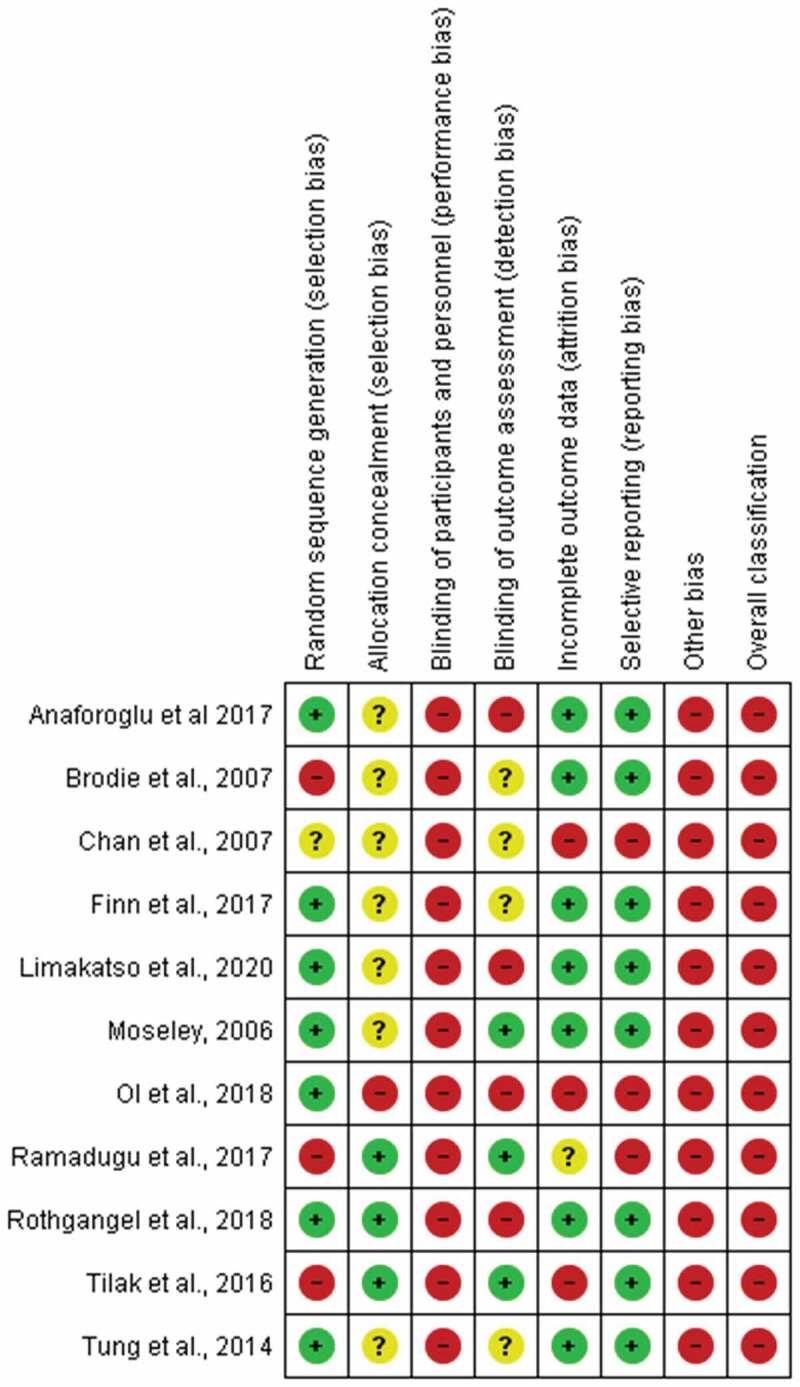
Authors’ judgments about each risk of bias item across all included studies.

**Figure 3.**
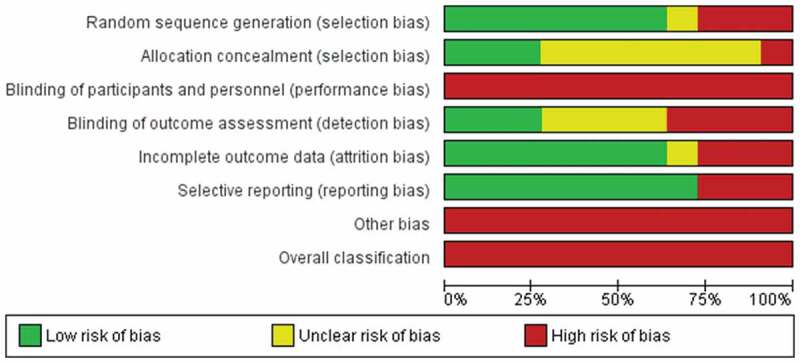
Authors’ judgments about each risk of bias item presented as percentages across all included studies.

The results of the risk of bias assessment are shown in Figures 2 and 3. All included studies had high risk of bias in the blinding category: no participants or treating clinicians were blinded to group allocation. In addition, only three studies^35,39,42^ were scored low risk for blinding outcome assessors to group allocation. All studies scored a high risk of additional bias for using an assessment tool that is not validated for assessing PLP in people with amputations. All of the studies scored a high risk for overall bias.

### Effects of the Interventions

#### Graded Motor Imagery Program

**Figure 4.**
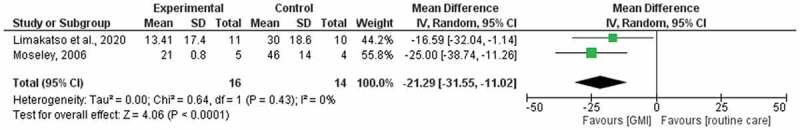
Forest plot for the effects of GMI versus routine care on pain severity at 6 weeks.

**Figure 5.**
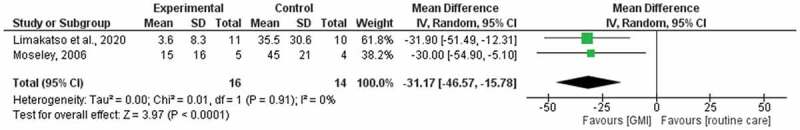
Forest plot for the effects of GMI versus routine care on pain severity at 6 months.

The pooling of two^6,35^ studies comparing GMI with routine care at 6 weeks generated a weighted mean difference of −21.29 (95% CI −31.55, −11.02) and low statistical heterogeneity (*I*^2^ = 0%; Fig. 4). The pooling of two studies comparing GMI with routine care at 6 months generated a median difference of −31.17 (95% CI −46.57, −15.78) and low statistical heterogeneity (*I*^2^ = 0%; Fig. 5). The quality of evidence according to the GRADE system is presented in Table 2. We found very low-quality evidence that GMI is effective and better than routine care for reducing PLP. The quality of evidence was downgraded by high risk of bias and a small sample size.

**Table 2.** The certainty of the evidence.

**Graded motor imagery versus routine care for phantom limb pain**				
**Population**: Adults (>18 years old) with phantom limb pain after limb amputation **Intervention**: Graded motor imagery **Comparison**: Routine care				
Outcome	Weighted mean difference (95% CI)	Number of participants (studies)	Confidence in effect estimate	Rating
Pain intensity (0–10 scale)	−21.29 (−31.55, −11.02)	30 (2)	⊕◯◯◯	Downgraded two levels; risk of bias and imprecision
**Mirror therapy versus covered mirror therapy for phantom limb pain**				
**Population**: Adults (>18 years old) with phantom limb pain after limb amputation **Intervention**: Mirror therapy **Comparison**: Covered mirror therapy				
Outcome	Weighted mean difference (95% CI)	Number of participants (studies)	Confidence in effect estimate	Rating
Pain intensity (0–10 scale)	−4.43 (−16.03, 7.16)	172 (4)	⊕◯◯◯	Downgraded two levels; risk of bias and imprecision
**Mirror therapy versus control treatments for phantom limb pain**				
**Population**: Adults (>18 years old) with phantom limb pain after limb amputation **Intervention**: Mirror therapy **Comparison**: Control treatments				
Outcome	Weighted mean difference (95% CI)	Number of participants (studies)	Confidence in effect estimate	Rating
Pain intensity (0–10 scale)	−13.12 (−15.65, −10.59)	146 (4)	⊕◯◯◯	Downgraded two levels; risk of bias and imprecision

Moseley’s^35^ comparison of a 6-week course of GMI to a 6-week course of routine physiotherapy also assessed pain-related disability using a patient-specific functional scale.^45^ Compared with routine physiotherapy, participants who performed GMI had less disability immediately after treatment (−2.40, 95% CI −0.82, −3.98). However, there was no between-group difference in disability scores at 6-month follow-up (0, 95% CI −1.68, 1.68).

Limakatso et al.’s^6^ comparison of a 6-week course of GMI to a 6-week course of routine physiotherapy also assessed HRQoL using the VAS of the EuroQol EQ-5D-5L.^45^ Compared with routine physiotherapy, participants who performed GMI had a higher HRQoL immediately after treatment (13.14, 95% CI −4.63, 30.91) and at 6-month follow-up (13.44, 95% CI −3.07, 29.95).

#### Mirror Therapy

The pooling of eight studies comparing mirror therapy with control treatments generated a weighted mean difference of −8.55 (95% CI −14.74, −2.35) but high statistical heterogeneity (*I*^2^ = 61%). Visual inspection of the funnel plot failed to suggest publication bias (Fig. 1 in Supplementary file 4). We found very low-quality evidence that mirror therapy is more effective than control treatments for reducing PLP (Table 2). The quality of evidence was downgraded by high risk of bias, a small sample size, and wide 95% CI.

**Figure 6.**
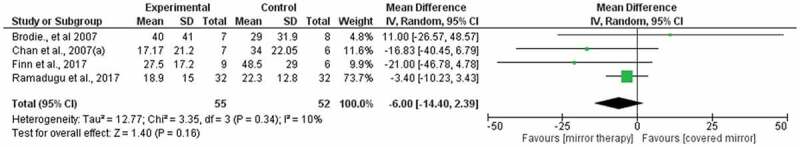
Forest plot for the effects of mirror therapy versus covered mirror therapy on pain severity.

**Figure 7.**
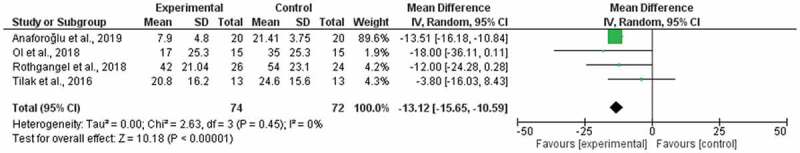
Forest plot for the effects of mirror therapy versus control treatments on pain severity.

A subgroup analysis of four studies^36–39^ comparing mirror therapy with covered mirror therapy generated a weighted mean difference of −4.43 (95% CI −16.03, 7.16) but high statistical heterogeneity (*I*^2^ = 51%; Fig. 6). A subgroup analysis of four studies^32,40–42^ comparing mirror therapy to other control treatments generated a weighted mean difference of −13.12 (95% CI −15.65, −10.59; Fig. 7) and low statistical heterogeneity (*I*^2^ = 0%). The certainty of the evidence was very low for both meta-analyses (Table 2). The quality of evidence was downgraded by high risk of bias, a small sample size, and a wide 95% CI.

#### Imagined Movements

Chan et al.’s^37^ comparison of imagined movements with covered mirror therapy showed a mean difference of −16.40 (95% CI −33.30, 0.50). Tung et al.’s^43^ comparison of imagined movements with movement observation showed a mean difference of 5.50 (95% CI −5.52, 16.52). Varying treatment protocols and control interventions in these studies meant that pooling them for a meta-analysis was not feasible.

## Discussion

The aim of this review was to systematically evaluate the effectiveness of GMI and its components on PLP in people with amputations. Our findings indicate that GMI was probably more effective than routine care for reducing PLP intensity immediately after treatment and at 6-month follow-up. The subgroup analyses based on the type of control treatment showed that mirror therapy was probably more effective than other control treatments but probably no more effective than covered mirror therapy for reducing PLP. The certainty of the evidence for studies evaluating GMI and mirror therapy was ranked as very low, primarily due to a high risk of bias and small sample size. Studies evaluating the efficacy of imagined movements showed no effect.

### Efficacy of GMI and Its Components on PLP

#### Graded Motor Imagery Program

Two studies^6,35^ provided evidence that GMI reduced PLP and pain-related disability. These studies corroborate the results of a retrospective case series^46^ that found that GMI had a clinically meaningful and long-lasting effect on PLP. GMI was endorsed in a recent expert Delphi study^47^ as a viable treatment for PLP, and the findings of this review further support its clinical utility. However, the generalizability of our findings is limited by a small sample size and a homogenous sample of lower limb amputees in the included studies. Replicating these positive findings in a large randomized, sham-controlled trial is necessary to shed light on the efficacy of GMI for reducing PLP in people with upper and lower limb amputations.

#### Mirror Therapy

Clinically significant pain reductions suggest that mirror therapy may be a more viable treatment for PLP compared to mental visualization techniques. These positive findings are consistent with those of preliminary studies that were not were not eligible for inclusion in the review.^48–53^ We found it interesting that one study^40^ that provided mirror therapy for a longer term showed superior effects compared to other studies. These findings are in line with expert recommendations that mirror therapy is likely to have a clinically meaningful effect when conducted at least three time a week over a long term.^17^ Moreover, our findings indicate that the efficacy of mirror therapy can be augmented by combining it with other treatments, as seen in GMI studies.^6,35^ The studies on mirror therapy included in this review had high risk of bias, and only one^40^ followed their participants beyond the immediate posttreatment assessment. This gap in the literature warrants robust clinical trials with a longer follow-up period and mechanisms-based studies to clearly elucidate the mechanisms by which mirror therapy reduces PLP.

Mirror visual feedback is argued to be an active component for mirror therapy.^18^ However, we found no difference in pain severity between mirror therapy and covered mirror therapy, for which mirror visual feedback is eliminated. Although visual input has been shown to influence phantom limb awareness,^54^ our results indicate that it is not necessary for PLP reduction. More recently, Ortiz-Catalan hypothesized that PLP is driven by the stochastic entanglement of somatosensory, motor, and pain networks resulting from somatosensory and motor deprivation.^16^ This concept implies that retraining somatosensory and motor networks, and not visual networks, is sufficient for pain reduction.

#### Imagined Movements

We found conflicting results for imagined movements in this review. An explanation for the conflicting results after imagined movements emerges from a consideration unique to the use of imagined movements in people with amputations. Raffin et al.^55,56^ pointed out that clinicians using imagined movements with people with intact limbs verify the absence of movement-generating neural activity by monitoring movement of the intact limb. However, such visual monitoring is not feasible in amputees, because the relevant body part is not present. It is therefore impossible to visually verify the absence of movement-generating neural activity in amputees participating in imagined movements, yet this verification is necessary, because any such activity would place a higher demand on the neural system than is desirable during imagined movements exercises.^56^

In line with this idea, Raffin et al.^55^ asked amputees with PLP to perform imagined movements, or movements of the phantom limb, while monitoring cortical activity, residual limb muscle activity (using electromyographic biofeedback), and sensations felt during the task. They found that performance of imagined movements was associated with neural activity in the premotor cortex and posterior lobe of the cerebellum, absent residual limb muscle activity and no triggered or exacerbated pain. In contrast, movements of the phantom limb were associated with neural activity in the primary somatosensory and primary motor cortices and the anterior lobe of the cerebellum, increased residual limb muscle activity, and, in some cases, pain exacerbation. These findings support the suggestion that conflicting findings across studies testing the efficacy of imagined movements for reducing PLP in amputees could be due to discrepancies in accurate performance of the imagined movements tasks; in other words, that participants asked to *imagine* performing movements are, in fact, performing movements, resulting in more neural activation than is desired.

#### Left/Right Judgments

We found no studies that evaluated left/right judgment exercises as a stand-alone treatment for PLP. Recent evidence suggests that left/right judgment exercises facilitate inhibitory priming of brain areas that are involved in preparation for movement.^57^ These data provide a logical rationale for the use of left/right judgment exercises as the first step in the GMI program, yet other studies^35,58^ that applied GMI to a group with mixed chronic pain conditions found left/right judgment exercises alone to have no effect on pain. There is a need to investigate the role of left/right judgment exercise in amputees with PLP.

### Strengths and Limitations

Our study had several strengths. We conducted this systematic review in accordance with a preregistered and published protocol. In addition, we followed the Cochrane recommendations for conducting a systematic review of controlled trials to ensure the robustness of the review process. A limitation of this review is that we excluded studies written in languages other than English, due to a lack of translation resources. The included studies represented few upper limb amputees, so the results may have limited generalizability to upper limb amputees. Only one study investigating mirror therapy followed up their participants beyond the time of treatment cessation. Therefore, we are unable to draw a firm conclusion on the long-term effects of this treatment on PLP.

In conclusion, this systematic review found weak but promising evidence that GMI is more effective than routine care for producing a clinically meaningful change in pain severity and that mirror therapy is more effective than mental visualization techniques but not covered mirror therapy. Importantly, our conclusion on GMI was derived from the results of few studies with a relatively small sample size. Higher quality studies are needed to generate a robust conclusion regarding the effectiveness of GMI on PLP.
